# ProNGF is a potential diagnostic biomarker for thyroid cancer

**DOI:** 10.18632/oncotarget.8652

**Published:** 2016-04-08

**Authors:** Sam Faulkner, Severine Roselli, Yohann Demont, Jay Pundavela, Genevieve Choquet, Philippe Leissner, Christopher Oldmeadow, John Attia, Marjorie M. Walker, Hubert Hondermarck

**Affiliations:** ^1^ School of Biomedical Sciences & Pharmacy, Faculty of Health and Medicine, University of Newcastle, Callaghan NSW 2308, Australia; ^2^ Hunter Medical Research Institute, University of Newcastle, New Lambton NSW 2305, Australia; ^3^ Inserm U908, Growth Factor Signaling and Functional Proteomics of Breast Cancer, University of Lille, 59655 Villeneuve d'Ascq, France; ^4^ Medical Diagnostic Discovery Department, bioMérieux, 69280 Marcy l'Etoile, France; ^5^ School of Mathematical and Physical Sciences, Faculty of Science and Information Technology, University of Newcastle, Callaghan NSW 2308, Australia; ^6^ School of Public Health & Medicine, Faculty of Health and Medicine, University of Newcastle, Callaghan NSW 2308, Australia; ^7^ Present address: INSERM U1138 team 11, Centre de Recherche des Cordeliers, 75006 Paris, France

**Keywords:** growth factors, proNGF, thyroid cancer, diagnostic biomarker

## Abstract

The precursor for nerve growth factor (proNGF) is expressed in some cancers but its clinicopathological significance is unclear. The present study aimed to define the clinicopathological significance of proNGF in thyroid cancer. ProNGF expression was analysed by immunohistochemistry in two cohorts of cancer *versus* benign tumors (adenoma) and normal thyroid tissues. In the first cohort (40 thyroid cancers, 40 thyroid adenomas and 80 normal thyroid tissues), proNGF was found overexpressed in cancers compared to adenomas and normal samples (p<0.0001). The area under the receiver-operating characteristic (ROC) curve was 0.84 (95% CI 0.75-0.93, p<0.0001) for cancers *versus* adenomas, and 0.99 (95% CI 0.98-1.00, p<0.0001) for cancers *versus* normal tissues. ProNGF overexpression was confirmed in a second cohort (127 cancers of various histological types and 55 normal thyroid tissues) and using a different antibody (p<0.0001). ProNGF staining intensity was highest in papillary carcinomas compared to other histological types (p<0.0001) and there was no significant association with age, gender, tumor size, stage and lymph node status. In conclusion, proNGF is increased in thyroid cancer and should be considered as a new potential diagnostic biomarker.

## INTRODUCTION

Thyroid cancer is a common malignancy with a rapidly increasing global incidence [1]. Although mortality from thyroid cancer is relatively low, the rate of disease recurrence or persistence is high, leading to increased patient morbidity and mortality [1]. Histological types of thyroid cancer include the relatively differentiated papillary, follicular and medullary cancers, as well as the undifferentiated anaplastic cancers. Aside from thyroid cancers, benign thyroid tumors (adenomas) represent the majority of clinically detected thyroid nodules. In clinical practice, microscopic examination of fine needle aspiration biopsy (FNAB) is the critical diagnostic test for evaluation of the cancerous nature of thyroid nodules. Unfortunately, in 10-15% of cases a definitive diagnosis cannot be made after FNAB, and the tumor is classified by the pathologist as “indeterminate” or “suspicious” [1]. No individual thyroid cancer biomarker has been found with sufficient sensitivity and specificity [2]. To resolve this diagnosis dilemma, new biomarkers of thyroid cancer are needed.

The precursor for nerve growth factor (proNGF) consists of the mature NGF polypeptide plus a propeptide of equivalent molecular mass at the N-terminus [3]. ProNGF can also generate NGF after processing by various proteolytic enzymes, such as furin or matrix metalloproteases [3]. However, proNGF also exhibits its own biological activities on neurons through the stimulation of specific receptors [3, 4]. ProNGF binds to the membrane protein sortilin [3, 4], a member of the Vacuolar Protein Sorting 10 protein (VPS10P) [5], to activate the neurotrophin receptor p75^NTR^ and the tyrosine kinase receptor TrkA (NTRK1) [3]. Original findings have described proNGF as an inducer of neuron apoptosis [6], but other studies have reported that its neurotrophic activities result in neuron survival and differentiation [7, 8]. These seemingly contradictory data can be explained by differential levels of sortilin, p75^NTR^ and TrkA at the neuronal cell surface, resulting in differential activations of downstream signaling pathways, such as those involving ERK, SRC or PI3K [9].

ProNGF is also expressed in some malignancies. In breast cancer, proNGF stimulates cancer cell invasion *via* the stimulation of TrkA and sortilin [10], and this tumor promoting effect is particularly relevant for the stem cell compartment of breast tumors [11]. In prostate cancer, proNGF expression correlates with aggressiveness and the growth of nerves into the tumor [12]. ProNGF also stimulates the invasion of melanoma cells through an interaction with p75^NTR^ and sortilin [13]. In thyroid cancer, oncogenic rearrangements of TrkA have been described, particularly in the histological type papillary carcinoma [14, 15]. TrkA as well as its TRK-T1 fusion protein induce neoplastic transformation of the thyroid epithelium [16, 17]. The expression of p75^NTR^ has also been reported in papillary thyroid carcinoma [18, 19]. Although NGF has been described in thyroid cancer, it has not been associated with any clinicopathological features [19]. In contrast, proNGF expression, to our knowledge, has not been reported.

In the present study, we aimed to determine the expression and clinicopathological significance of proNGF in thyroid cancer. The expression of proNGF was analyzed by immunohistochemistry in two cohorts of cancers *versus* normal thyroid tissues. The data show that proNGF is overexpressed in thyroid cancer and therefore could constitute a novel biomarker for diagnosis.

## RESULTS

### ProNGF expression in cohort 1: comparison of thyroid cancers *versus* adenomas and normal tissues

**Figure 1:**
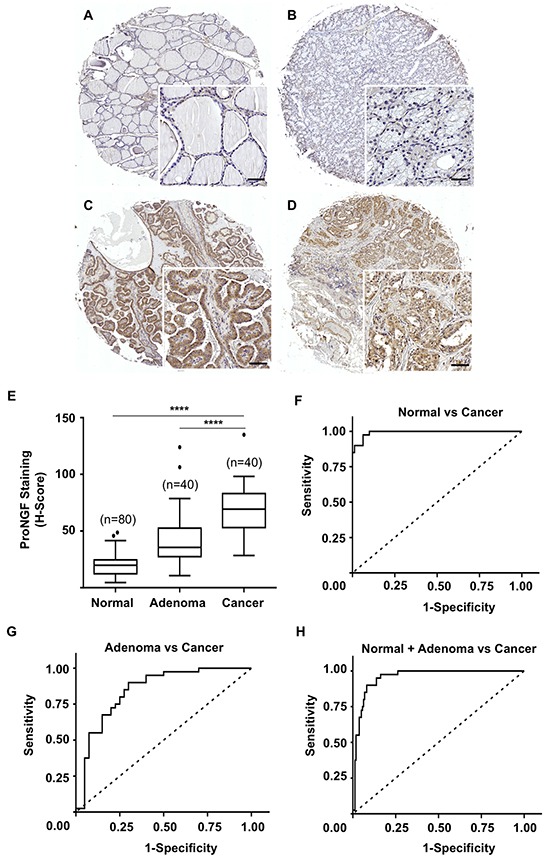
ProNGF expression in cohort 1 of thyroid cancers versus adenomas and normal tissues **A–D.** Immunohistochemical detection of proNGF was performed with a polyclonal antibody on a series of thyroid cancers (n=40), adenoma (n=40) and normal thyroid tissues (n=80). ProNGF was found in epithelial cells with a marked increased in cancer tissues. Representative pictures are shown for normal thyroid tissue (A), adenoma (B), papillary carcinoma (C), follicular carcinoma (D). Scale = 50μm. **E.** Quantification of proNGF staining intensities was performed using the Halo™ image analysis platform, h-scores were calculated and used to establish the ROC curves. ProNGF staining intensities were significantly higher for cancers (median h-score = 69.3) than adenomas (median h-score = 35.5) and normal tissues (median h-score = 19.7) (p<0.0001). The box limits indicate the 25th and 75th percentiles with the whiskers extending 1.5 times the interquartile range from the 25th and 75th percentiles (outliers are represented by dots) (****p<0.0001). **F–H.** ROC curves for proNGF staining intensity levels in thyroid cancers versus adenomas and normal thyroid tissues were established and analyzed using GraphPad™. The area under the curve was 0.99 (95% CI 0.98-1.00, p<0.0001) for cancers versus normal samples (F), 0.84 (95% CI 0.75-0.93, p<0.0001) for cancers versus adenomas (G), and 0.95 (95% CI 0.85-0.96, p<0.0001) for cancers versus adenomas and normal samples (H).

ProNGF expression was investigated by immunohistochemistry, using a polyclonal antibody, in a series of 40 cases of thyroid cancer, 40 adenomas and 80 normal thyroid tissues. ProNGF was detected in epithelial cells with a marked increase in cancers compared to normal tissue (Figure 1A–1D). No labeling was observed in the stroma (including endothelial cells, fibroblasts and the extracellular matrix). Digital quantification of staining intensities (Figure 1E) revealed a median h-score of 19.7 in normal thyroid tissues, 35.5 in adenomas and 69.3 in cancer (p<0.0001). The corresponding ROC curves are presented (Figure 1F–1H). The area under the ROC curve (AUROC) of cancer *versus* normal tissue (Figure 1F) was 0.99 (95% CI 0.98-1.00, p<0.0001). Similarly, the AUROC of cancers *versus* adenomas (Figure 1G) was 0.84 (95% CI 0.75-0.93, p<0.0001). When considering cancers *versus* adenomas + normal tissue (Figure 1H), the AUROC was 0.95 (95% CI 0.92-0.98, p<0.0001). For analysing the correlations between proNGF expression and clinicopathological parameters, proNGF staining intensities were categorized as 0 (h-score <25), 1 (h-score 25-50), 2 (h-score 50-75), and 3 (h-score >75). The frequency distribution is presented in Table 1 and indicated that 100% of thyroid cancer (including papillary, follicular and medullary histological types) were positive for proNGF as compared to 87% adenoma and 22% of normal tissues (p<0.0001). Importantly, the proportion of samples with intermediate and high levels of proNGF (staining intensities 2 and 3) shifted from 0% in normal tissues to 27% in adenomas and 82% in cancer. The analysis of association with clinicopathological parameters indicated that 96% of papillary carcinomas presented high levels (staining intensities 2 and 3) of proNGF compared to 88% of follicular carcinomas and 17% of medullary carcinomas (p=0.0006). Overall and for each histological type, there was no association between proNGF expression and other clinicopathological parameters (age, gender, tumor size, stage and lymph node status).

**Table 1:** ProNGF expression in thyroid cancers versus adenomas and normal tissues (cohort 1) and associations with clinicopathological parameters

Parameters	ProNGF Intensity				*p*-value
	0	1	2	3	
***Normal vs. Cancer***					**< 0.0001**
Normal (n=80)	62 (78%)	18 (22%)	0 (0%)	0 (0%)	
Adenoma (n=40)	5 (13%)	24 (60%)	8 (20%)	3 (7%)	
Cancer (n=40)	0 (0%)	7 (18%)	17 (42%)	16 (40%)	
***Clinical Parameters in Cancers***					
***Histological Type***					**0.0006**
Follicular (n=8)	0 (0%)	1 (12%)	5 (63%)	2 (25%)	
Papillary (n=26)	0 (0%)	1 (4%)	11 (42%)	14 (54%)	
Medullary (n=6)	0 (0%)	5 (83%)	1 (17%)	0 (0%)	
***Gender***					0.9952
Female (n=30)	0 (0%)	5 (17%)	13 (43%)	12 (40%)	
Male (n=10)	0 (0%)	2 (20%)	4 (40%)	4 (40%)	
***Age (Years)***					0.4385
<50 (n=24)	0 (0%)	4 (17%)	8 (33%)	12 (50%)	
≥50 (n=16)	0 (0%)	3 (19%)	9 (56%)	4 (25%)	
***Tumour Size (T)***					0.9254
T1 + T2 (n=14)	0 (0%)	3 (21%)	5 (36%)	6 (43%)	
T3 + T4 (n=26)	0 (0%)	4 (15%)	12 (46%)	10 (39%)	
***Lymph Node Status***					0.9055
Negative (n=37)	0 (0%)	6 (16%)	16 (43%)	15 (41%)	
Positive (n=3)	0 (0%)	1 (33%)	1 (33%)	1 (33%)	
***Stage***					0.6106
I + II (n=25)	0 (0%)	4 (16%)	9 (36%)	12 (48%)	
III + IV (n=15)	0 (0%)	3 (20%)	8 (54%)	4 (26%)	

Pro NGF immunohistochemical staining in each sample was quantified and categorized as 0 = no staining (h-score <25), 1 = low staining (h-score 25-50), 2 = intermediate staining (h-score 50-75), 3 = strong staining (h-score >75). Representative pictures and corresponding ROC curves are presented in Figure 1. For each category, the number of cases is indicated, and the corresponding percentage is under brackets. Statistically significant p-values (p<0.05 using chi-square test) are shown in bold and were confirmed in Log linear analysis.

### ProNGF expression in cohort 2: comparison of thyroid cancer of different histological types and normal tissues

**Figure 2:**
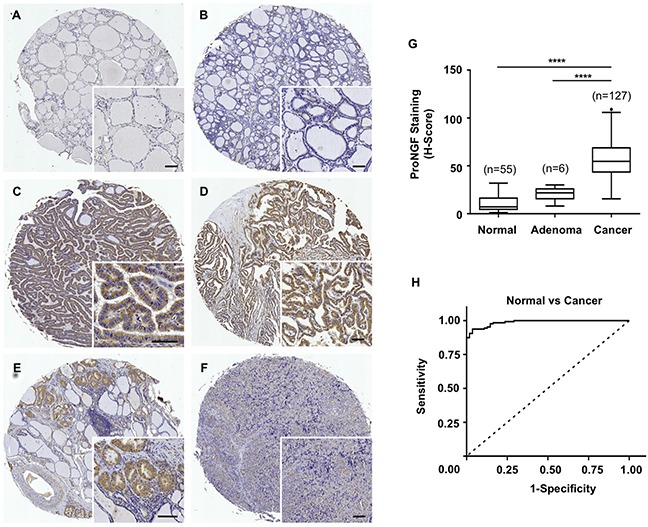
ProNGF expression in cohort 2 of thyroid carcinomas of different histological types *versus* normal tissues **A-F.** Immunohistochemical detection of proNGF in thyroid cancers of various histological types (n=127 cases), adenomas (n=6) and normal thyroid tissues (n=55) was performed witha monoclonal antibody. ProNGF was found in epithelial cells with a marked increased in cancer tissues. Representative pictures are shown for normal thyroid tissue (A), adenoma (B), papillary carcinoma (C), follicular carcinoma (D), medullary carcinoma (E), and anaplastic carcinoma (F). Scale = 50μm. **G.** Quantification of proNGF staining intensities was performed using the Halo™ image analysis platform, h-scores were calculated and used to establish the ROC curves. ProNGF staining intensities were significantly higher for cancers (median h-score = 54.6) than adenomas (median h-score = 21.7) and normal tissues (median h-score = 7.2). The box limits indicate the 25th and 75th percentiles with the whiskers extending 1.5 times the interquartile range from the 25th and 75th percentiles (outliers are represented by dots) (****p<0.0001). **H.** The ROC curve for proNGF staining intensity levels in thyroid cancers *versus* normal thyroid tissues was established and analyzed using GraphPad™. The area under the ROC curve was 0.98 (95% CI 0.97-0.99, p<0.0001).

ProNGF expression was analysed by immunohistochemistry, using a monoclonal antibody, in a series of 127 thyroid cancers of various histological types, 6 adenomas and 55 normal thyroid tissues. ProNGF was preferentially detected in thyroid cancer cells and rarely in normal thyroid tissues (Figure 2A–2F). ProNGF staining appeared in all tumor types including papillary, follicular, medullary and anaplastic carcinomas. Digital quantification of staining intensities (Figure 2G) confirmed the overexpression of proNGF in cancers as observed in cohort 1. The median h-score for proNGF staining was 7.2 in normal thyroid tissues, 21.7 in adenomas and and 54.6 in cancers (p<0.0001). The difference in h-score was not different between normal and adenomas, but was significantly different between cancer and adenomas (p<0.0001). The ROC curve of cancer versus normal (Figure 2H) indicated an AUROC of 0.98 (95% CI 0.97-0.99, p<0.0001). There were not enough adenoma cases (n=6) in this cohort to draw a relevant ROC curve for proNGF intensities between adenomas and cancer. The categorization of proNGF staining intensities is presented in Table 2. High levels of proNGF (staining intensities 2 and 3) were found in 60% of thyroid cancer, particularly in the papillary and follicular types, as compared to 0% of normal tissue and adenoma samples (p<0.0001). Considering the distribution of proNGF in the different histological types of thyroid cancers, papillary carcinomas presented with 74% of high proNGF staining intensities (staining intensities 2 and 3) compared to 34% of follicular carcinomas and 25% of anaplastic carcinomas (p<0.0001). This association of proNGF expression with papillary histological types was confirmed in Log linear analysis (p=0.0098), controlling for stage and gender. In addition, the odds of papillary relative to follicular cancer were increased by a factor of 2.07 for increased proNGF level (p=0.039). There was evidence of crude associations of proNGF expression with gender (p=0.004), but this was not confirmed in Log linear analysis. No significant associations (p<0.05) were found between proNGF expression and other clinicopathological parameters (age, tumor size, stage and lymph node status).

**Table 2:** ProNGF expression in thyroid cancers of different histological types (cohort 2) and associations with clinicopathological parameters

Parameter	ProNGF Intensity				*p*-value
	0	1	2	3	
***Normal vs. Cancer***					**< 0.0001**
Normal (n=55)	53 (96%)	2 (4%)	0 (0%)	0 (0%)	
Adenoma (n=6)	5 (83%)	1 (17%)	0 (0%)	0 (0%)	
Cancer (n=127)	8 (6%)	43 (34%)	53 (42%)	23 (18%)	
***Clinical Parameters in Cancers***					
***Histological Type***					**< 0.0001**
Follicular (n=26)	1 (4%)	16 (62%)	7 (26%)	2 (8%)	
Papillary (n=79)	1 (1%)	20 (25%)	38 (49%)	20 (25%)	
Anaplastic (n=12)	5 (42%)	4 (33%)	3 (25%)	0 (0%)	
Others (n=10)	1 (10%)	3 (30%)	5 (50%)	1 (10%)	
***Gender***					**0.0004^#^**
Female (n=100)	2 (2%)	35 (35%)	41 (41%)	22 (22%)	
Male (n=27)	6 (22%)	8 (30%)	12 (44%)	1 (4%)	
***Age (Years)***					0.8519
<50 (n=74)	4 (5%)	24 (33%)	31 (42%)	15 (20%)	
≥50 (n=53)	4 (8%)	19 (35%)	22 (42%)	8 (16%)	
***Tumour Size (T)***					0.0929
T1 + T2 (n=29)	2 (7%)	6 (21%)	15 (51%)	6 (21%)	
T3 + T4 (n=59)	4 (7%)	28 (47%)	21 (36%)	6 (10%)	
Missing (n=39)	2 (5%)	9 (23%)	17 (44%)	11 (28%)	
***Lymph Node Status***					0.3055
Negative (n=68)	6 (9%)	24 (35%)	27 (40%)	11 (16%)	
Positive (n=17)	0 (0%)	9 (53%)	7 (41%)	1 (6%)	
Missing (n=42)	2 (5%)	10 (24%)	19 (45%)	11 (26%)	
***Stage***					0.1041
I + II (n=54)	1 (2%)	22 (41%)	22 (41%)	9 (16%)	
III + IV (n=34)	5 (15%)	12 (35%)	14 (41%)	3 (9%)	
Missing (n=39)	2 (5%)	9 (23%)	17 (44%)	11 (28%)	

Pro NGF immunohistochemical staining in each sample was quantified and categorized as 0= no staining (h-score <25), 1 = low staining (h-score 25-50), 2 = intermediate staining (h-score 50-75), 3 = strong staining (h-score >75). Representative pictures and corresponding ROC curves are presented in Figure 2. For each category, the number of cases is indicated and the corresponding percentage is under brackets. Statistically significant p-values (*p*<0.05 using chi-square test) are shown in bold and were confirmed in Log linear analysis for normal vs cancer and histological types.

# The association with gender was not confirmed in Log linear analysis.

## DISCUSSION

This study reports for the first time the expression of proNGF in thyroid cancer, and has shown a potential value of this growth factor as a biomarker to differentiate thyroid cancer from adenoma and normal thyroid. The molecular pathogenesis of thyroid cancer remains to be clarified, but abnormalities in key signaling pathways have been described [20]. Genetic and epigenetic alterations in thyroid tumors include mutations (BRAF^V600E^, Ras, PI3K, PTEN, p53, b-catenin, anaplastic lymphoma kinase), translocation (RET-PTC) and paired box 8 (PAX8)-peroxisome proliferator-activated receptor-g (PPARG) as well as aberrant gene methylation (retinoic acid receptor beta, and tissue inhibitor of metalloprotease 3) [20]. Gene amplifications and copy-number gains have also been described, particularly for genes encoding receptor tyrosine kinases such as EGFR, VEGFR, KIT, MET and PDGF [20]. Interestingly, gene rearrangements of the neurotrophin receptor TrkA have also been reported to play a role in thyroid tumor progression [21, 14, 15, 16]. Although the molecular alterations described so far in thyroid cancer provided opportunities for clinical development as biomarkers and therapeutic targets, their clinicopathological significance has not been demonstrated. Molecular markers such as RAS, BRAF, PAX8/PPAR**c**, RET/PTC, may be considered for indeterminate cytology according to American Thyroid Association guidelines [22]. However, the American Association of Clinical Endocrinologists and the European Thyroid Association do not currently recommend these markers in routine practice but reserved them for selected cases due to theirs inconsistent results and relatively high costs [23]. In this context, our study demonstrating the overexpression of proNGF in thyroid cancer points to the potential clinical utility of a novel and reliable growth factor as a new diagnostic biomarker. A microquantification of proNGF could eventually be applied to fine needle aspirates, and help to categorize the indeterminate/suspicious samples, but this warrants further technical investigations. The accurate preoperative diagnosis of thyroid cancer continues to be a significant challenge. No individual thyroid cancer biomarker has been found with sufficient sensitivity and specificity [2]. However, a panel comprised of GAL3, CK19 and HBME1 is by far the most studied to date and offers some improvement over individual marker performance alone [2]. In the future, it would be important to evaluate the diagnostic performance of proNGF in comparison and combination with the currently used biomarkers.

In terms of gene expression, NGF mRNA abundance has not been reported to be linked to a particular clinicopathological parameter in thyroid cancer. Before investigating proNGF protein levels by immunohistochemistry, we have performed a data mining of NGF gene expression, using cBioportal [24], of thyroid datasets in The Cancer Genome Atlas (TCGA) database [25]. NGF mRNA upregulation was found in only 23 out of 507 patient cases, representing only 5% of the total number of cases. Also, a point mutation (K153R) was found in one single patient. No cases of NGF mRNA downregulation were detected. Initial studies in yeast have suggested a correlation of about 50% between mRNA and protein levels [26], and in humans, global transcriptomic and proteomic analyses have shown that only an estimated 30% of changes in protein levels can be explained by corresponding variations in mRNA [27]. Interestingly, a recent proteogenomic investigation in colorectal cancer has also revealed that mRNA abundance does not reliably predict differences in tumoral protein levels [28]. This emphasizes the importance of analysing proteins directly in cancer tissue, to define new biomarkers and therapeutic targets in oncology.

ProNGF expression has been reported in melanoma [13], breast [10] and prostate [10] cancers. In melanoma and breast cancer, proNGF stimulates invasion of cancer cells [10, 12], whereas in prostate cancer, proNGF participates in nerve infiltration into the tumor [12]. Interestingly, in breast and prostate cancers, higher levels of proNGF were reported in malignant tumors compared to benign tissues [10, 12]. Therefore, proNGF overexpression appears to be a feature of several cancers, including thyroid cancer as demonstrated in the present study. The use of two different antibodies (polyclonal and monoclonal) further strengthens the demonstration that proNGF is overexpressed in thyroid cancer. Unspecific cross-reactivity of antibodies is a potential pitfall of IHC. Not only we have performed the necessary negative controls, but also the same overexpression of proNGF was observed using the two antibodies, and this is reassuring that it is indeed specific to proNGF. In addition, we have not detected any association between proNGF expression and the presence of nerve fibers in thyroid tumors. Nerve fibers were seen in less than 5% of thyroid cancers and this was independent of proNGF expression (data not shown). Therefore, in contrast to prostate cancer [12], the expression of proNGF in thyroid cancer is not related to nerve infiltration. In the nervous system, proNGF binds to a complex between sortilin and p75^NTR^ or TrkA, depending on the relative receptor concentrations at the cell surface [2, 29]. In thyroid cancer, TrkA expression and gene rearrangements have been reported [21, 14, 15, 30] and given the tyrosine kinase activity of TrkA, it participates in the deregulation of thyroid cancer cell growth [16, 17]. It has also been shown that p75^NTR^ is widely expressed in papillary thyroid carcinoma [18] and sortilin is expressed in thyroid epithelial cells, where it contributes to the recycling of the thyroid hormone precursor thyroglobulin [31]. However, the determination of a biological activity for proNGF has not been investigated here. Also additional questions arise, such as the effects of proNGF expression on therapeutic and external accidental ionizing radiation of the thyroid. Together, further *in vitro* and *in vivo* experiments are warranted to define a possible function for this growth factor in thyroid cancer progression.

In conclusion, this study demonstrates an increased level of proNGF in thyroid cancers and suggests that this growth factor has potential as a new diagnostic biomarker. In addition, as proNGF is a secreted protein, its potential value as a blood biomarker should also be considered. Together, further investigations to assess the impact and clinical utility of proNGF in thyroid cancer are warranted.

## MATERIALS AND METHODS

### Thyroid tissue samples

High-density tumor microarrays (TMA, TH801, TH802, TH804, TH641, TH8010) were obtained from Biomax (Maryland, USA). Cohort 1 included 40 thyroid cancers (26 papillary, 8 follicular, 6 medullary) (TH802), 40 adenomas (TH802) and 80 normal thyroid glands (TH804). Cohort 2 included 127 thyroid cancers (79 papillary, 26 follicular, 12 anaplastic, 10 from other histological subtypes) (TH801, TH641, TH8010), 6 adenomas (TH641) and 55 normal thyroid tissues (TH801, TH8010). Cohort 2 also included the following clinicopathological information: patient age and sex, histological type, tumor size, lymph node status and stage. No information on treatment and patient survival was available. The small core size (1.5 mm diameter) and the bias introduced by sampling is a general limitation of using TMAs. Biomax (USA) quality controls are described as follows. Each single tissue spot on every array slide is individually examined by pathologists certified according to WHO published standardizations of diagnosis, classification and pathological grade. Pathological re-confirmation report is generated and digital image captured. Standard immunohistochemistry tests are also performed to ensure the accuracy and specificity of tissue array products. Each specimen collected from any clinic was consented to by both hospital and individual. Discrete legal consent form was obtained and the rights to hold research uses for any purpose or further commercialized uses were waived. The study was approved by the Human Research Ethic Committee of the University of Newcastle, Australia.

### Immunohistochemistry

After deparaffinization and rehydration of TMA slides following standard procedures, heat induced epitope retrieval was carried out in a low pH, citrate based antigen unmasking solution (Vector Laboratories, California, USA, catalogue number H-3300) using a decloaking chamber (Biocare, West Midlands, United Kingdom) at 95°C for 20min. After inactivation of endogenous peroxidases with 0.3% H_2_O_2_, and blocking with 2.5% horse serum, anti-proNGF antibodies were applied to the sections and revealed with DAB Peroxidase (HRP) Substrate Kit (Vector Laboratories, California, USA, catalogue number SK-4100). Two different anti-proNGF antibodies were used. A polyclonal anti-proNGF antibody (Merck Millipore, Darmstadt, Germany, catalogue number AB9040) was used at 1/200 for cohort 1, and a monoclonal anti-proNGF antibody made against the proNGF propeptide sequence (in house from Biomerieux, Marcy l'Etoile, France) was used at 2.5 μg/ml for cohort 2. Specific controls of the used antibodies are shown in Supplementary Figure S1. In Western-blotting both the polyclonal and monoclonal antibodies recognized proNGF but not NGF (Supplementary Figure S1A). In addition, negative controls with no primary antibodies or control isotype antibodies (Mouse (G3A1) mAb IgG1 Isotype Control, Cell Signaling Technology, Massachusetts, USA, catalogue number #5415) were also performed (Supplementary Figure S1B, C). TMA slides were also counterstained with hematoxylin (Gill's formulation, Vector Laboratories, California, USA), dehydrated and cleared in xylene before mounting in Ultramount #4 mounting media (Thermo Fisher Scientific, Victoria, Australia). Imaging was performed on an Axioplan-2 microscope (Carl Zeiss AG, Oberkochen, Germany).

### Digital quantification of immunohistochemistry

For quantification of proNGF staining, TMA slides were digitized at 200x absolute resolution using an Aperio AT2 scanner (Leica Biosystems, Victoria, Australia). Quantitative IHC analyses were performed using the Halo™ image analysis platform (Indica Labs, New Mexico, USA) under the supervision of a pathologist (MMW). The pixel intensities of DAB staining were calculated using the Area Quantification algorithm. Pixel intensity values were then used to determine the h-scores for each core (index calculated as the sum of 3 x % of pixels with strong staining + 2 x % of pixels with intermediate staining + 1 x % pixels with weak staining). To compare proNGF levels across the cohort, the h-scores were used to divide cases into 4 categories (0 = h-score <25, 1 = h-score 25-50, 2 = h-score 50-75, 3 = h-score >75).

### Statistical analyses and determination of associations with clinicopathological parameters

The staining intensity for proNGF was compared with clinicopathological parameters: normal *versus* malignant, patient age and gender, histological type, tumor size, stage, lymph node status. For statistical analysis, simple unadjusted associations with pathological variables were performed using a chi-squared test. We used log-linear models to adjust the various bivariate associations for other potential confounders. The log linear models provided a Chi-squared test adjusted for all other variables in the model. The model for the cross-classified counts was specified as a Poisson generalised linear model with a log-link function. Using hierarchical nesting of models we looked at all 3-way then 2-way interactions involving proNGF intensity (modelled as an ordinal variable). Goodness of fit was tested using G^2^ Chi-squared statistics (comparing the log likelihood to that obtained from the saturated model). Nested models were compared by calculating differences in *G*^2^ statistics and were used to assess removal (a non-significant reduction in fit) of terms to the model. The prognostic value of the biomarker was expressed using the area under the receiver-operating characteristic (AUROC) curve; values close to 0.5 indicate performance close to chance, while values close to 1 indicate near perfect discrimination. These models were fitted using SAS (SAS Institute, North Carolina, USA).

## SUPPLEMENTARY FIGURE



## Abbreviations

AUROC: area under the receiver-operating characteristic curve
CI: confidence interval
FNAB: fine needle aspiration biopsy
NTRK1: neurotrophin receptor tyrosine kinase 1
p75^NTR^: p75 neurotrophin receptor
NGF: nerve growth factor
proNGF: NGF precursor
TMA: tumor microarray
