# Supplementary material for: ProNGF is a potential diagnostic biomarker for thyroid cancer
Source: Oncotarget. 2016 Apr 8;7(19):28488–97. doi: 10.18632/oncotarget.8652 (PMC5053740; doi:10.18632/oncotarget.8652)
Supplement: Supplementary file 1 [file oncotarget-07-28488-s001.pdf]

SUPPLEMENTARY FIGURE

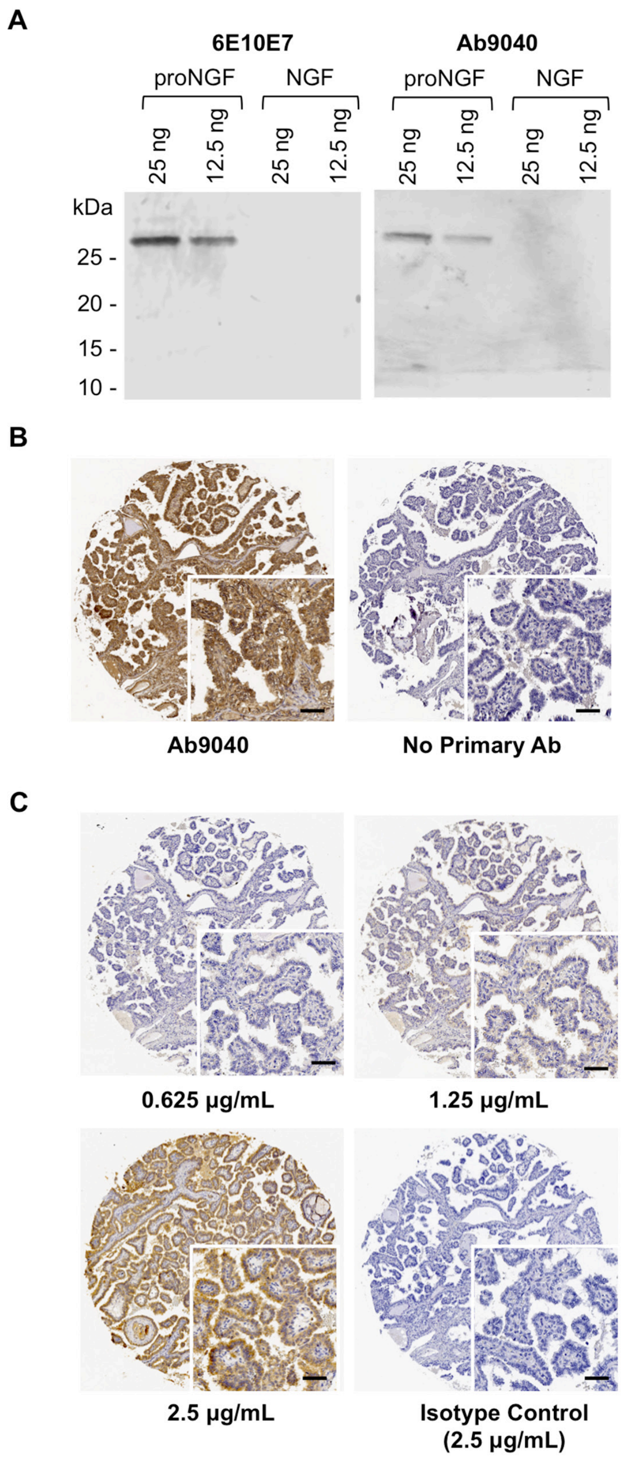

Supplementary Figure S1: Specific controls for proNGF antibodies. A polyclonal anti-proNGF (Ab9040) and a monoclonal anti-proNGF (6E10E7) were tested in Western-blotting and immunohistochemistry. **A.** Western-blotting was performed against proNGF and NGF. Two different quantities per lane (25 ng and 12.5 ng) were used for proNGF and NGF. The two antibodies recognized proNGF but not NGF. **B.** Negative control with no primary antibody in immunohistochemistry (with a papillary thyroid cancer) for the Ab9040 antibody. **C.** Dilution of the 6E10E7 antibody in immunohistochemistry (0.625, 1.25 and 2.5 µg/mL with a papillary carcinoma) and negative control using an isotype control antibody at the concentration of 2.5 µg/mL.
